# Insights Into the Expectations of Infertile Men Regarding Multidisciplinary Reproductive Health Services

**DOI:** 10.1111/hex.70327

**Published:** 2025-07-01

**Authors:** Mehrdad Abdullahzadeh, Zohreh Vanaki, Eesa Mohammadi, Jamileh Mohtashami

**Affiliations:** ^1^ Department of Nursing, Faculty of Medical Sciences Tarbiat Modares University Tehran Iran; ^2^ Department of Psychiatric Nursing, School of Nursing and Midwifery Shahid Beheshti University of Medical Sciences Tehran Iran

**Keywords:** couple‐centred care, healthcare providers, male infertility, multidisciplinary treatment, nursing skills, qualitative research, reproductive health services

## Abstract

**Aim:**

This qualitative study sheds light on the expectations of infertile men regarding reproductive health services.

**Design:**

This descriptive qualitative study employed an inductive content analysis approach.

**Methods:**

In 2023, nurse researchers conducted in‐depth interviews with 13 men with primary infertility using a semi‐structured approach. The collected data were analysed using Graneheim and Lundman's inductive content analysis method, adhering to Guba and Lincoln's standards to ensure trustworthiness.

**Results:**

The analysis identified the central theme, ‘Male Infertility: Awareness, Support and Participation,’ as well as three categories: Male Infertility Awareness, which involves spreading the word in various ways; Male Infertility Support, encompassing all professional expertise; and Male Infertility Programme, which involves couples' joint participation in treatment.

**Conclusions:**

Men seeking reproductive health services necessitate a comprehensive treatment approach involving education, consultations with specialists and couple‐centred care. This finding highlights the importance of multidisciplinary reproductive health services, which can help healthcare providers, nurses and policymakers enhance the quality of care for men and couples with male infertility. Adopting a multifaceted approach incorporating nursing skills and competencies can promote more inclusive and culturally sensitive patient‐centred care in reproductive health services.

**Patient or Public Contribution:**

Individuals with lived experiences analysed and interpreted data.

## 1 Introduction

Reproductive health services are critical for the sexual and reproductive well‐being of both men and women and encompass various medical and healthcare services, such as family planning, prenatal care, screening and treatment for sexually transmitted infections (STIs), counselling on sexual and reproductive health issues and infertility treatment [1]. However, men's reproductive health needs have often been overlooked or undervalued, resulting in inadequate attention being given to their unique needs [2]. This gender disparity in care provision has created a significant demand for specialized reproductive health services that address men's specific needs [3].

## 2 Background

Infertility affects 17.5% of adults worldwide, irrespective of income level or region, according to a report by the World Health Organization (WHO) [4]. Although the causes of female infertility are diverse and no separate estimates exist, male infertility contributes to 50% or more of infertility cases. This is the primary issue in 30% of such cases. Since 1990, the prevalence and rate of male infertility have increased by 76.9% and 19%, respectively [5].

Male infertility is a prevalent and complex issue that can have significant impacts on individuals, partners and society at large. These impacts can be physical, psychological, social and economic, leading to feelings of inadequacy, frustration and depression [6]. Despite the prevalence of male infertility, many countries and regions worldwide lack adequate and accessible reproductive health services for men [7]. This is because reproductive health services often focus on women's needs and perspectives, while men's roles and experiences are usually neglected or marginalized [8].

Many infertile men face barriers when seeking and receiving reproductive healthcare, such as stigma, lack of awareness, low health literacy, cultural norms, gender stereotypes, financial constraints and poor quality of care [9, 10]. A systematic review and meta‐synthesis by Roudsari et al. [11] identified four main barriers to men's participation in reproductive healthcare: failure to access all‐inclusive and integrated quality services, economic issues, couples' personal preferences and attitudes and sociocultural considerations. Geng et al. [12] stated that these obstacles are shaped by healthcare system plans and policies, as well as economic and sociocultural factors and men's attitudes, knowledge and preferences. These conditions can prevent men from receiving appropriate reproductive care and lead to delays in the diagnosis, treatment and management of their condition.

A holistic and integrated approach to male reproductive health is necessary to address the physical, psychological, social and economic aspects of male infertility [13]. This approach recognizes the importance of involving men in reproductive healthcare decisions [14]. Healthcare providers should prioritize developing practical strategies that centre on the patient to enhance men's reproductive health outcomes and overall well‐being [15]. To improve their approaches, address the barriers to accessing medical care and encourage men to prioritize reproductive health, reproductive health services need to understand the needs and experiences of men with infertility [16].

## 3 The Study

To ensure optimal care for male reproductive health, it is crucial to address the gaps in care provision for men's needs [17]. Men should have access to comprehensive reproductive health services, including regular checkups and screenings for STIs, sexual performance, sexual health and fertility [18]. Understanding men's expectations regarding reproductive health services is crucial for developing effective interventions that meet their needs as a human right [19, 20]. To determine effective care strategies, this article provides insights into infertile men's expectations regarding reproductive health services.

## 4 Methods

### 4.1 Design

We conducted this qualitative study with a critical approach, grounded in the nursing metaparadigm, which affirms that the person's health, environment and nursing are necessary for care [21]. Our manuscript adheres to the Consolidated Criteria for Reporting Qualitative Research (COREQ) [22]. The COREQ checklist is available in Supporting File 1.

### 4.2 Study Setting and Recruitment

Nurse researchers conducted this study from March to November 2023 at a public fertility centre in Isfahan, Iran. The researchers employed purposive sampling to recruit male participants with diverse characteristics, including different types of infertility, a range of ages, geographical locations in Iran, and various treatment methods. Male participants were identified by staff at the fertility centre. They met specific eligibility criteria to be included in the study, such as confirmation of their infertility from a fertility specialist, primary male infertility related solely to the male (not female), and no history of drug addiction in the past 12 months. These criteria excluded factors affecting male reproductive health [23].

### 4.3 Data Collection

The research team's first author was a PhD candidate and a male registered nurse who did not participate in the care of the study participants. The author approached participants who were eligible for the study and provided them with an invitation letter explaining the study's objective. The invitation emphasized that participation was voluntary and confidential. The participants signed a consent form and were interviewed in a private room at the fertility centre by the first author, who was also the interviewer. All interviews were semi‐structured and focused on male infertility following diagnosis. The participants were asked about their experiences with reproductive health services, including their expectations of such services. Additional questions, such as ‘Can you tell me more?’ or ‘What do you mean?’ were asked. The interviews were audio‐recorded digitally and transcribed verbatim immediately after each interview by the interviewer. The participants spoke in Farsi, and the first author provided English translations. The translations were then reviewed and backtranslated to Farsi to preserve their meaning. During the interviews, a partner was present for one participant at their request but was inactive in the discussion. At the end of the interview, the interviewer asked if the participants would like to add anything. Further counselling assistance was also provided if needed.

We conducted 15 interviews with 13 participants. Two participants were interviewed twice for clarification. Saturation was confirmed after 15 interviews, including two follow‐ups, when three consecutive interviews yielded no new codes or categories during iterative coding sessions, and no new themes could be identified, as participant responses became redundant. Therefore, the collected data can be considered comprehensive and reflective of the phenomenon under investigation.

### 4.4 Data Analysis

We employed qualitative inductive content analysis techniques, as described by Graneheim and Lundman [24]. The recursive process involved multiple steps, from reading each interview transcript several times to understanding each participant's story and account. Meaningful units, such as words, sentences and paragraphs, were identified and condensed without losing content. Each condensed unit was given a code that summarized its content, and these codes were grouped to form subcategories and categories. Categories and central themes were derived based on interpretations of meaning in the classes. Citations, clarifications and quotations were used to exemplify the results. The analysis was performed manually using the cut‐and‐paste method. The nurse authors held critical meetings throughout the study to reflect on and review the interpretations of the findings and handle coding differences. The research groups had diverse backgrounds, including clinical and reproductive healthcare counselling, as well as experience in qualitative research.

### 4.5 Rigour and Reflexivity

The researchers implemented several measures to ensure the reliability and trustworthiness of the study results. We adhered to Lincoln and Guba's standards and invited an experienced professor as an external researcher to review the anonymized transcripts [25]. The researchers meticulously documented every step of the research process to ensure that the findings could be easily transferred and replicated. The researchers accurately recounted the participants' words without alteration or modification and returned the transcripts to them for comment and correction to maintain authenticity. We established a friendly and engaging relationship with the participants throughout the research process, creating a conducive environment that encouraged open and honest feedback. This allowed for a more robust and comprehensive analysis of the findings. Finally, the researchers made a conscious effort to present the information in an engaging and informative manner, ensuring that the participants' contributions were accurately represented and valued.

### 4.6 Ethical Considerations

The Ethics Committee of Tarbiat Modares University approved this study (Ethic no: IR.MODARES.REC.1401.212). During the study, the interviewer informed the participants about the purpose and duration of the research, addressed any concerns they may have had, and provided written informed consent forms for their review. The researchers also offered psychiatric consultations to the participants in case of need and ensured their anonymity by providing them with unique secret code numbers. All the procedures carried out in the study, which involved human participants, complied with the ethical standards of the institutional and national research committee, as well as with the 2013 Helsinki Declaration or similar ethical standards.

## 5 Results

### 5.1 Characteristics of the Participants

The study involved 13 individuals, including men with primary infertility. The interviews lasted between 20 and 90 min. Demographic details are presented in Table 1.

**Table 1:** Characteristics of male participants.

Alias	Age (year)	Being with the spouse (years)	Age gap with spouse (years)	Time trying to conceive (years)	Position	Education	Interview duration (min)	Number of interviews
1	30	4	4	2	Self‐employed	Diploma	60	1
2	30	10	4	9	Self‐employed	Bachelor's	70	1
3	34	10	4	5	Employee	Diploma	50	1
4	40	10	4	9	Employee	Diploma	60	1
5	36	6	6	6	Employee	Primary school	20	1
6	37	12	1	6	Self‐employed	Diploma	80	1
7	30	4	1	4	Self‐employed	Diploma	45	1
8	38	11	5	9	Employee	Primary school	75	2
9	39	10	12	9	Employee	Primary school	55	1
10	41	6	2	6	Employee	Masters'	45	1
11	29	4	1	4	Self‐employed	Primary school	65	1
12	40	13	2	13	Employee	Bachelor's	70	1
13	33	8	5	3	Employee	Bachelor's	90	2
Mean ± SD	35.15 ± 4.22	8.30 ± 3.04	3.92 ± 2.84	6.53 ± 2.97	—	—	60.38 ± 17.37	—

### 5.2 Qualitative Findings

**Figure 1:**
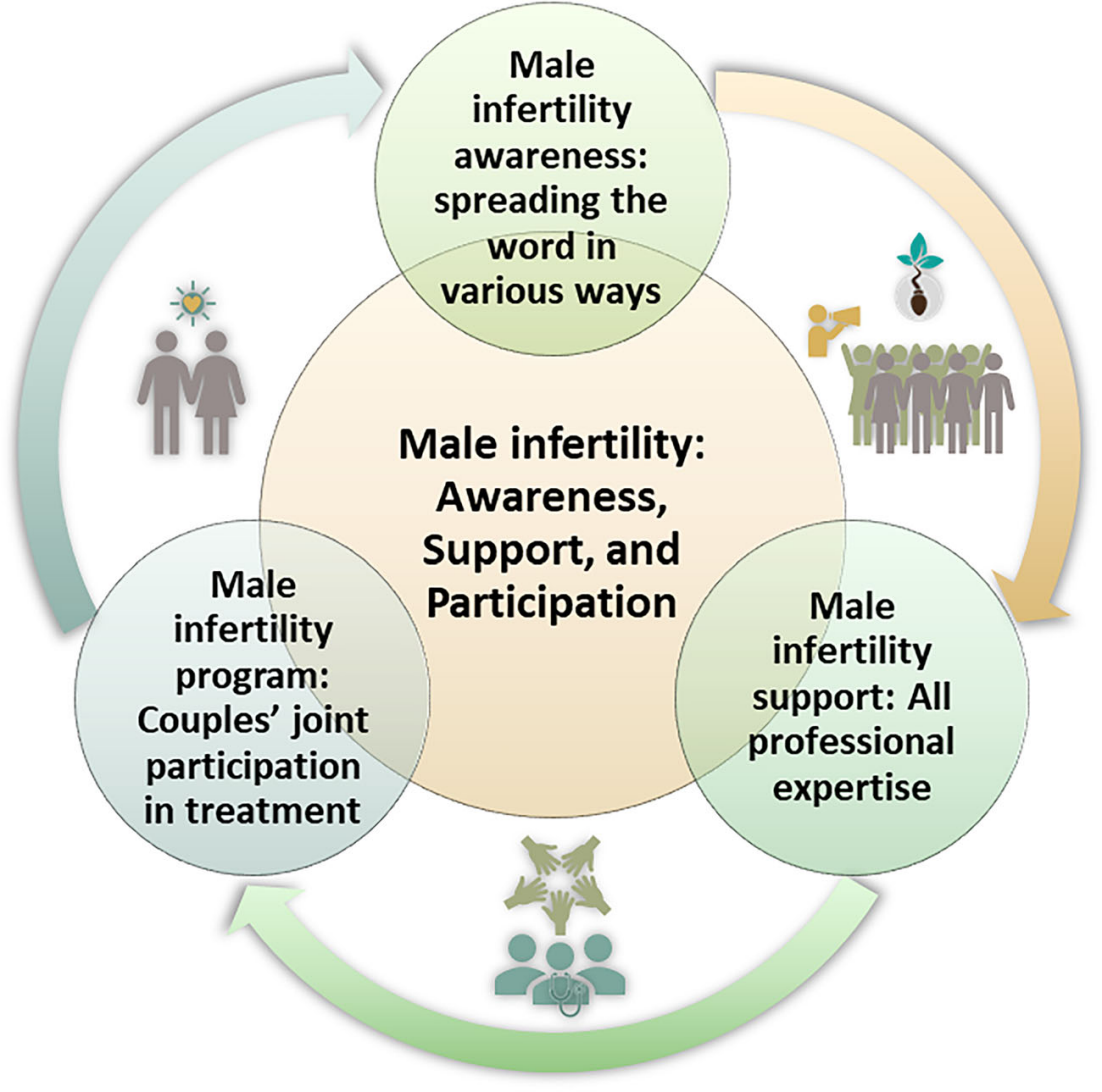
Conceptual model of the central theme ‘Male Infertility: Awareness, Support and Participation.’ Arrows indicate interconnections between categories: (1) awareness campaigns to address misconceptions, (2) multidisciplinary professional support and (3) couple‐centred care promoting joint involvement.

Our data analysis revealed a central theme, which was categorized into three distinct categories. An abstract model was created to facilitate the interpretation of the findings (Figure 1). We included direct participant quotes to illustrate these categories. The results, track, confirmation and condensation codes are presented in Supporting File 2.

#### 5.2.1 Male Infertility: Awareness, Support and Participation

The main topic of the conversation is ‘Male Infertility: Awareness, Support and Participation,’ which encompasses essential principles across various categories. This concept reflects the complexity of men's expectations regarding multidisciplinary reproductive health services. The central theme highlights the importance of raising awareness about male infertility and available treatment options, providing comprehensive consultations involving multiple disciplines to address male infertility, and developing couple‐centred care plans. Several factors can shape these expectations, including healthcare system policies, economic and sociocultural influences, and men's attitudes, knowledge and preferences.

#### 5.2.2 Male Infertility Awareness: Spreading the Word in Various Ways

Our research indicates that men seeking reproductive health services are often uninformed about male infertility and the available treatment options. This lack of awareness is influenced by cultural perceptions that equate a man's ability to engage in intercourse and masculinity with fertility, while often framing infertility as solely a woman's issue. Such misconceptions can lead to delayed diagnosis and treatment, resulting in further complications and emotional distress. For instance, we encountered a case in which a man was diagnosed with male infertility despite having no prior knowledge of the condition:

I was ignorant about male infertility, and I was too anxious to get help until I determined that I had it. At first, I blamed my wife for not having children, but she was okay.

(P3)

Men experiencing infertility sought information on the causes, symptoms, diagnosis and treatment options, and they emphasized the need to raise awareness of this issue among men as follows:

I have always wondered why this happened. For example, I heard among friends that someone had an accident, and then they said that they could not have children because of the accident. When we first married, I thought it was a woman's problem because I did not know about male infertility. At that time, I kept asking my wife, “Why don't you have children?” Maybe it was my mistake. When we went for medical tests, the doctors found no problem with my wife. After they asked me to take a test, I realized my problem was with me. (Reproductive health) Services should increase people's knowledge about male infertility and the treatments available.

(P10)

They believe that addressing male infertility should be a shared responsibility between partners and that both couples should receive comprehensive and inclusive reproductive health education on male infertility:

It is crucial to raise awareness about male infertility so that couples can seek appropriate treatment. Unfortunately, many partners fail to follow up on treatment because of a lack of understanding. When we were informed of our infertility, it was either dismissed or not recognized as a problem. For example, my wife tended to assume that a man's ability to engage in intercourse and masculinity are indicators of good sperm quality. However, this is not necessarily true. Even if a man appears to have healthy sexuality, there may still be abnormalities that could result in infertility.

(P12)

#### 5.2.3 Male Infertility Support: All Professional Expertise

The feedback we received from the participants highlights the necessity for reproductive services to provide all‐encompassing consultations that involve multiple disciplines to tackle the issue of male infertility. The participants sought guidance and assistance regarding various male infertility treatment options available to them. For instance, one male participant felt that he did not receive adequate support or explanations regarding the treatment procedure, which resulted in him not following up with his treatment:

A doctor advised me to undergo a testicular tissue sample, but they did not explain it to me in detail. I had scheduled an appointment, but I was anxious and scared to go through with it. I was unaware of the procedure and was worried that it might be painful. As a result, I did not go through with it. More information and education should be provided regarding the various treatments available to patients.

(P4)

The consultation process should adopt a holistic approach, considering all aspects of the patient's medical history, lifestyle, educational background, cultural influences, legal implications and other relevant factors contributing to infertility. For example, one participant shared his experiences in this way:

In addition to treatments, I may need a psychiatrist or psychologist who has expertise in these fields to help me—someone who understands the types of problems a person like me may face. There must be experts to support me in terms of treatment costs and the legal issues that may exist regarding the acceptance of donated sperm or embryos.

(P9)

The participants stated that the involvement of multiple healthcare professionals can help identify the causes of male infertility and develop appropriate treatment plans tailored to each patient's individual needs.

Sometimes, I feel frustrated and wish to have someone else listen to and advise me. I may also need other experts to help me handle this problem (Male infertility). They should understand that each person faces unique challenges in this situation.

(P13)

#### 5.2.4 Male Infertility Programme: Couples' Joint Participation in Treatment

The participants discussed the importance of couple‐centred care for male infertility. They stated that male infertility can affect both partners' feelings and thoughts and that couple‐centred care can help them deal with such problems and enhance their relationship. A man told his story about how his wife helped him cope with infertility difficulties:

My wife has been an unwavering source of support throughout my life. Her presence has been invaluable in resolving the challenges of infertility. Although she was under pressure, whenever I felt discouraged, she would remind me that there was always a solution to every problem.

(P6)

Another man discussed the important role his wife played in supporting him throughout the treatments:

Her unwavering optimism and enthusiasm motivated me even when specific treatments were ineffective. She ensured that we followed the treatment plan and regularly inquired about doctors and medicines.

(P8)

They emphasized that male infertility can have a significant impact on the emotional and mental well‐being of both partners and that couple‐centred care can help address the underlying issues and improve communication and intimacy.

I was unhappy when I learned that I was infertile. However, my wife comforted me and said we could try treatment, embryo donation, or adoption. She said that many people have this problem and encouraged me. She told me to hope. We had two options: trust God or find alternatives. Her words moved her, but she supported me in my therapy. I wish there were more help for women with infertile male partners. The treatment program should allow both partners to help each other.

(P11)

## 6 Discussion

This qualitative study aimed to shed light on the expectations of infertile men regarding reproductive health services. The main finding was that multidisciplinary treatment for couples is an integral part of reproductive health services for infertile men. Our study's findings have revealed an urgent need for a more holistic and inclusive approach to male fertility awareness. Schlegel et al. [26] stated that the reproductive education approach should cover all aspects of fertility, including male infertility and the various available treatments. Research by Carson and Kallen [27] indicated that such an approach would significantly enhance the quality of reproductive health services and contribute to the overall well‐being of individuals and families. Based on Cervi and Knights [28], it is essential to prioritize the development and implementation of evidence‐based interventions and knowledge that effectively address issues related to male infertility. Reproductive health services should be accessible to everyone, regardless of gender, sexual orientation, or financial status. These services must also be practical and culturally sensitive. Therefore, by promoting awareness and understanding of male infertility and considering sociocultural factors, reproductive health services can play a significant role in creating an informed and empowered society.

According to our study, reproductive health services should adopt a multidisciplinary and comprehensive approach to consultations for male infertility. Eisenberg et al. [29] stated that male infertility is a complex medical condition that requires a comprehensive strategy involving multiple disciplines. Bhattacharya et al. [30] reported that evaluating and treating male infertility requires expertise from various fields. Healthcare professionals must adopt a comprehensive and collaborative approach involving all required disciplines. Ravindran and Govender [31] indicated that a holistic and personalized treatment plan that considers various disciplines can improve reproductive health services' outcomes, including conception and overall reproductive health. Moreover, healthcare professionals should consider their patients' cultural and religious beliefs while working together to provide the best possible care.

Our research emphasizes the significance of recognizing male infertility as a mutual responsibility between partners rather than a problem that only affects men. As mentioned by Bergman and Petok [32], male infertility can have multiple causes and consequences for couples, and addressing these complexities is crucial for providing effective reproductive health services. Shreffler et al. [33] recommended that couple‐centred care, early intervention and access to specialized care are vital to increasing the chances of successful treatment and improving overall outcomes for struggling couples with male infertility. Agarwal et al. [34] reported that infertility can be a challenging experience. Effective communication and mutual support can help couples navigate the challenges of male infertility. Healthcare professionals can provide a safe space for partners to share their feelings and concerns, thereby facilitating emotional support. By prioritizing open communication and mutual support, healthcare services can help couples not only cope better with male infertility but also develop a deeper connection and stronger relationship.

### 6.1 Strengths and Limitations of Work

This study explores the insights and expectations of infertile men regarding multidisciplinary reproductive health services. The findings can contribute to the development of effective interventions, support services and patient‐centred care for men undergoing infertility treatment. However, certain biases may affect the results, including selection bias due to purposive sampling, cultural specificity arising from a focus on a single country, and social desirability bias inherent in self‐reported data. Although efforts were made to address these limitations by recruiting a diverse sample, the findings apply only to a specific group of male patients with infertility and cannot be generalized to all individuals facing similar health issues. In addition, some participants may have felt hesitant or uncomfortable communicating with the researchers, which could have influenced the study outcomes. Therefore, it is essential to consider these limitations and nuances when interpreting the findings of this study.

### 6.2 Implications for Research, Policy and Practice

This study underscores the importance of conducting qualitative research to better understand how infertile men perceive reproductive health services across different cultural and social settings. Examining the factors that shape these expectations is equally important. In Iran, deeply ingrained societal beliefs linking male fertility to virility often contribute to delayed diagnoses and reinforce the stigma around infertility. Overcoming these challenges calls for culturally sensitive strategies, including community‐driven awareness initiatives featuring religious leaders. Furthermore, comparative studies across diverse settings could offer deeper insights into how sociocultural norms shape care‐seeking behaviours. To reduce gender disparities in reproductive health, policymakers should integrate male infertility care into existing healthcare programmes by training providers and utilizing telemedicine and digital platforms to expand accessibility. Broader access to reproductive health services can also be achieved through community outreach programmes, collaborative public–private efforts and financial assistance frameworks that make care more affordable. Emphasizing couple‐centred care will lead to a more holistic, inclusive and effective approach to infertility treatment. Healthcare professionals should be attuned to the cultural expectations and barriers that infertile men encounter when accessing reproductive health services. They should focus on raising awareness about male infertility and available treatments, facilitating multidisciplinary consultations and promoting couple‐centred care. Additionally, providers should strive to create a more welcoming and supportive environment for men seeking reproductive health services, considering economic and sociocultural factors along with men's attitudes, knowledge and preferences.

## 7 Conclusion

This study examines the expectations of men with infertility regarding reproductive health services, highlighting the need for a multidisciplinary approach that educates affected individuals about male infertility and the available treatments. Men benefit from consulting multiple specialists and receiving couple‐centred care, reinforcing the importance of tailored reproductive health services that address their specific needs and experiences. The findings contribute to the existing literature by emphasizing the necessity of specialized, multidisciplinary reproductive health services, guiding healthcare providers, policymakers and nurses in improving the quality and inclusivity of care for men and couples dealing with male infertility. Cultural norms surrounding masculinity, fertility and healthcare‐seeking behaviours influence how men engage with reproductive health services, thus shaping the demand for multidisciplinary care. Socioeconomic factors such as healthcare access, affordability, and educational background affect men's awareness of infertility treatments and their ability to seek specialized consultations. Systemic factors—including healthcare policies, insurance coverage, and the availability of reproductive health specialists—vary across regions, impacting the feasibility of implementing patient‐centred and couple‐focused care models. Recognizing these contextual differences is essential for adapting male infertility interventions to diverse populations and ensuring that reproductive health services remain inclusive, accessible and culturally sensitive. To achieve optimal care outcomes, healthcare providers should prioritize patient‐centred and inclusive approaches by developing culturally relevant strategies that focus on the patient while acknowledging the importance of involving men in reproductive healthcare decisions. A multifaceted approach that integrates nursing expertise and competencies can enhance patient‐centred care, ultimately improving reproductive health outcomes and overall well‐being.

## Author Contributions

**Mehrdad Abdullahzadeh:** conceptualization, investigation, funding acquisition, writing – original draft, methodology, validation, visualization, writing – review and editing, software, formal analysis, project administration, data curation, resources. **Zohreh Vanaki:** conceptualization, methodology, validation, visualization, formal analysis, project administration, supervision. **Eesa Mohammadi:** conceptualization, methodology, validation, visualization, formal analysis, supervision. **Jamileh Mohtashami:** methodology, formal analysis, validation, visualization. All authors have reviewed and licensed this version for publication.

## Ethics Statement

The Ethics Committee of Tarbiat Modares University approved this study (Ethic no: IR.MODARES.REC.1401.212).

## Consent

Participants provided written permission before each interview, and only those who gave their consent were included in the study. Participation was voluntary, anonymity was guaranteed and withdrawal did not affect the quality of healthcare or social support.

## Conflicts of Interest

The authors declare no conflicts of interest.

## Supporting information

S1.

S2.

## Data Availability

The datasets analysed during the current study are available from the corresponding author upon reasonable request.
