# Supplementary material for: Insights Into the Expectations of Infertile Men Regarding Multidisciplinary Reproductive Health Services
Source: Health Expect. 2025 Jul 1;28(4):e70327. doi: 10.1111/hex.70327 (PMC12210042; doi:10.1111/hex.70327)
Supplement: Supplementary file 1 — S1. [file HEX-28-e70327-s002.docx]

# **Supplementary 1:** Consolidated criteria for reporting qualitative studies (COREQ): 32-item checklist

| **No** | **Item** | **Guide questions/description** | **Page No** |
| --- | --- | --- | --- |
| **Domain 1: Research team and reflexivity** | | | |
| Personal Characteristics | | | |
| 1. | Interviewer/facilitator | Which author/s conducted the interview or focus group? | 6 |
| 2. | Credentials | What were the researcher's credentials? E.g., PhD, MD | 6 |
| 3. | Occupation | What was their occupation at the time of the study? | 6 |
| 4. | Gender | Was the researcher male or female? | 6 |
| 5. | Experience and training | What experience or training did the researcher have? | 6 |
| Relationship with participants | | | |
| 6. | Relationship established | Was a relationship established prior to study commencement? | 6 |
| 7. | Participant knowledge of the interviewer | What did the participants know about the researcher? e.g., personal goals, reasons for doing the research | 6 |
| 8. | Interviewer characteristics | What characteristics were reported about the interviewer/facilitator? e.g., Bias, assumptions, reasons, and interests in the research topic | 6 |
| **Domain 2: study design** | | | |
| Theoretical framework | | | |
| 9. | Methodological Orientation and Theory | What methodological orientation was stated to underpin the study? e.g., grounded theory, discourse analysis, ethnography, phenomenology, content analysis | 6 |
| Participant selection | | | |
| 10. | Sampling | How were participants selected? e.g., purposive, convenience, consecutive, snowball | 6 |
| 11. | Method of approach | How were participants approached? e.g., face-to-face, telephone, mail, email | 6 |
| 12. | Sample size | How many participants were in the study? | 7 |
| 13. | Non-participation | How many people refused to participate or dropped out? Reasons? | 7 |
| Setting | | | |
| 14. | The setting of data collection | Where was the data collected? e.g., home, clinic, workplace | 7 |
| 15. | Presence of non-participants | Was anyone else present besides the participants and researchers? | 7 |
| 16. | Description of sample | What are the important characteristics of the sample? e.g., demographic data, date | 9 |
| Data collection | | | |
| 17. | Interview guide | Were questions, prompts, and guides provided by the authors? Was it pilot-tested? | 7 |
| 18. | Repeat interviews | Were repeat interviews carried out? If yes, how many? | 7 |
| 19. | Audio/visual recording | Did the research use audio or visual recording to collect the data? | 7 |
| 20. | Field notes | Were field notes made during and/or after the interview or focus group? | 7 |
| 21. | Duration | What was the duration of the interviews or focus groups? | 7 |
| 22. | Data saturation | Was data saturation discussed? | 7 |
| 23. | Transcripts returned | Were transcripts returned to participants for comment and/or correction? | 8 |
| **Domain 3: analysis and findings** | | | |
| Data analysis | | | |
| 24. | Number of data coders | How many data coders coded the data? | 9 |
| 25. | Description of the coding tree | Did the authors provide a description of the coding tree? | 9 |
| 26. | Derivation of themes | Were themes identified in advance or derived from the data? | 7 |
| 27. | Software | What software, if applicable, was used to manage the data? | Non |
| 28. | Participant checking | Did participants provide feedback on the findings? | 8 |
| Reporting | | | |
| 29. | Quotations presented | Were participant quotations presented to illustrate the themes/findings? Was each quotation identified? e.g., participant number | 10-12 |
| 30. | Data and findings consistent | Was there consistency between the data presented and the findings? | 10-12 |
| 31. | Clarity of major themes | Were major themes clearly presented in the findings? | 9 |
| 32. | Clarity of minor themes | Is there a description of diverse cases or a discussion of minor themes? | 9-14 |
