# Supplementary material for: Insights Into the Expectations of Infertile Men Regarding Multidisciplinary Reproductive Health Services
Source: Health Expect. 2025 Jul 1;28(4):e70327. doi: 10.1111/hex.70327 (PMC12210042; doi:10.1111/hex.70327)
Supplement: Supplementary file 2 — S2. [file HEX-28-e70327-s001.docx]

**Supplementary 2.** The track and confirmation of findings, and the condensation codes, categories, and the central theme

| **Condensation Codes** | **Categories** | **Theme** |
| --- | --- | --- |
| There is a need to raise awareness about male infertility through various means. | Male infertility awareness: Spreading the word in various ways | Male infertility: Awareness, support, and participation |
| There is a need to raise awareness about male infertility treatments in different ways. |  |  |
| The need to be informed about the legal rules of reproductive assistance methods. |  |  |
| There is a need to be informed about the genetic problems of using reproductive assistance methods. |  |  |
| The necessity of counseling in the field of embryo donation and sperm donation. | Male infertility support: All professional expertise |  |
| The necessity of providing a specific program to confront couples with male infertility treatments. |  |  |
| There is a need to provide counseling about the psychological aspects of accepting a donated embryo before embryo transfer. |  |  |
| It is necessary to consult on blood group matching tests of the fetus and the recipient parents before embryo transfer. |  |  |
| There is a need to provide counseling about the genetic aspect of accepting a donated embryo before embryo transfer. |  |  |
| There is a need to receive legal advice about reproductive assistance methods. |  |  |
| There is a need to provide information about the donor embryo's genetic parents to the recipient couples. |  |  |
| The necessity of strict supervision of the law on embryo donation. |  |  |
| There is a need to carefully examine the eligibility of couples receiving donated embryos to take care of a child. |  |  |
| There is a need to carefully examine the mental competence of couples to accept donated embryos. |  |  |
| There is a need to continue the treatment process with both couples. | Male infertility program: Couples’ joint participation in treatment |  |
| There is a need for couples to visit medical professionals at the same time. |  |  |
| Couples must visit a psychiatrist at the same time. |  |  |
| There is a need for both couples to be involved in the treatment process. |  |  |
